# Factors contributing to the high prevalence of intimate partner violence among south Sudanese refugee women in Ethiopia

**DOI:** 10.1186/s12889-023-16343-x

**Published:** 2023-07-24

**Authors:** Filmawit Hadush, Dereje Tsegaye, Sherif Abdulwehab Legass, Endegena Abebe, Sabit Zenu

**Affiliations:** 1 Gender Coordinator at the Plan International, Gambella, Ethiopia; 2 Sexual and Reproductive Health Coordinator at International Medical Corps, Aysaista, Ethiopia; 3 Department of Public Health, College of Health Sciences, Mattu University, Mettu, Ethiopia; 4 Department of Biomedical Sciences, College of Health Sciences, Mattu University, Mettu, Ethiopia

**Keywords:** Intimate partner violence, Violence against women, Pinyudo, Refugees, Ethiopia

## Abstract

**Background:**

Intimate partner violence is a universally occurring form of violence against women which is perpetrated by a husband or other intimate partner. It is a common public health problem during humanitarian crisis. Despite this, little is known about the problem among South Sudanese refugee women in Ethiopia.

**Objective:**

This study aimed to determine the prevalence of intimate partner violence and identify its contributing factors among married refugee women in Pinyudo refugee camp, Gambella, Ethiopia in 2021.

**Methods:**

A community-based cross-sectional study was conducted from March to June 2021. A random sample of 406 refugee women was included in the study. A structured, pretested, and interviewer-administered questionnaire was used to collect the data. Data were entered into epi-data version 3.1 and exported to SPSS version 22 for analysis. Multivariable logistic regression was run to identify factors associated with intimate partner violence. Statistical significance was affirmed using Adjusted Odds Ratio with its 95% Confidence Interval at a *p*-value ≤ 0.05.

**Results:**

A total of 406 married refugee women participated in the study making a response rate of 96.2%. The overall prevalence of intimate partner violence in the past 12 months was 48.3% 95% CI= (43.6–53.2). Low-income contribution [AOR = 2.4, 95% CI: 1.2–5.5], and attitudinal acceptance [AOR = 2.1, 95%CI: 1.2–3.8] were significantly associated with the problem.

**Conclusion:**

The prevalence of intimate partner violence is alarmingly high as half of participating women reported facing the problem in the year preceding the study. Low-income contribution and attitudinal acceptance were associated with a higher probability of experiencing violence. The government, humanitarian organizations, and other stakeholders should enable refugee women to generate income. There should be continuous women empowerment and behavioral interventions to improve refugee women’s attitudes towards intimate partner violence.

## Introduction

Intimate partner violence (IPV) is one of the most common forms of violence against women (VAW), and includes physical, sexual, and emotional abuse as well as controlling behaviors by an intimate partner. It is a widespread, yet understudied social and health problem that occurs worldwide and worsen during times of crisis [1, 2].

According to the World Health Organization’s report of 2021, nearly 307 million partnered women suffered from IPV in the year preceding the study. Concerning region specific estimates, IPV in 12 months is most prevalent in least developed countries(22%). Sub-Saharan Africa (20%) and Southern Asia (19%) have the next highest prevalence rates of past 12 months IPV, followed by Northern Africa (15%) and Western Asia(13%) [2]. Studies showed the continued existences of IPV among partnered women in Ethiopia, where nearly half of such women experienced the problem in a year [3–6].The recent report from the Ethiopian Demographic and Health Survey(EDHS) found the prevalence of lifetime IPV among partnered Ethiopian women to be 34%. The 12 month prevalence of IPV among these women was 27% [7].

In humanitarian settings, as a result of mass displacement and the breakdown of social protections, refugee women are at increased risk of IPV [8]. The WHO considers the scarcity of reliable prevalence data on IPV among refugees and women in humanitarian settings as a significant challenge [2]. Efforts have been made to capture the prevalence of IPV among refugees in different areas of the world. Findings from South East Asia showed a very high prevalence of IPV among refugee women reaching as high as 80% among Afghan refugees in Iran [9], and 72% among Rohingya refugees in Bangladesh [10]. The magnitude is also high among Congolese refugees in Rwanda, where nearly 50% of women experienced IPV [11]. The figure is relatively lower among Eritrean refugees in Tigray Region of Ethiopia where 25% of refugee women experienced IPV [12].

IPV has significant short, medium, and long-term effects on health and wellbeing of women, children, and families. In addition to injuries and physical damage, victims and survivors are more likely to suffer from moderate to severe mental health outcomes in their post-violence life [13–15]. It also results in several untoward sexual and reproductive health consequences [16, 17]. IPV during pregnancy may cause miscarriage, premature labor, fetal injury, stillbirth, and low birth weight [2, 18–20]. Furthermore, it has a negative impact on the health of children causing anxiety, depression, poor school performance, and other undesirable health outcomes [18, 21–23]. Evidence also indicates that exposure to IPV against the mother is one of the most common factors associated with male perpetration and female experience of IPV later in life [24, 25]. Studies also found a significant co-occurrence of IPV and child abuse within the same household [18, 26].

Causes and risk factors of IPV are summarized as individual, relationship, and community and societal factors [18]. Women face an exacerbated form of all risk factors of IPV during times of humanitarian crisis and refugee conditions [27]. Refugees are the most vulnerable population to almost all health problems, especially to sexual and gender-based violence (SGBV) [28]. In some settings, women who experience physical and economic abuses inflicted by armed actors also experience a significant increase in IPV. In summary, refugee women face the double burden of IPV, especially in protracted displacement conditions [29, 30].

Several initiatives have been implemented to prevent the problem of GBV among refugee women. The United Nations Higher Commissioner for Refugees (UNHCR) first introduced a guideline to prevent GBV among refugees in 1995 [2]. Despite these efforts, the trend of IPV over the last decades showed a mixed trend, with a marked increase in some settings [31–33].

South Sudan violence has resulted in one of the biggest refugee crisis in Africa. Uganda, Sudan, Ethiopia, Kenya, the Democratic Republic of Congo and the Central African Republic continue to host over 2.2 million South Sudanese refugees. According to the latest report, Ethiopia is hosting 392,482 South Sudanese refugees in seven refugee camps in Gambella Regional State [34, 35].

The quantification of IPV is always difficult due to the nature of the problem and so more during times of crisis and in refugee settings. Only one piece of research has been conducted among refugees in Ethiopia to quantify the extent of IPV [12]. Research on Somali refugees used only qualitative assessment and failed to show the prevalence of the problem [36]. No published report has been found on the problem among South Sudanese refugees in Ethiopia. This study assessed the prevalence of IPV and its associated factors among married South Sudanese refugee women in Pinyudo refugee camps in South West Ethiopia.

## Materials and methods

### Study area, design, and period

This study was conducted among South Sudanese refugee women in Pinyudo refugee camps. The camp, divided as Pinyudo 1 and Pinyudo 2, is the oldest South Sudanese refugee camp in Ethiopia. Currently, the total population of the camp is estimated at around 52,346, and women account for 58% of the population. The refugees are provided with basic services like food, water, health care, education, shelter and protection. UNHCR and other partners provide relief services in collaboration with ARRA. A community-based cross-sectional study was conducted from March to June 2021.

### Sample size determination and sampling technique

The sample size was calculated for both objectives. The sample size for the first objective, prevalence of IPV, is calculated by using the single population proportion formula on STATCALC of Epi info version 7.2.0.1. The sample size for the second objective, factors associated with IPV, is calculated by using the double population proportion formula on the same software. The sample size for the first objective resulted in a higher sample size with a total sample size of 422 married refugee women. This sample size was calculated using the 95% Confidence Interval(CI), a margin of error of 5%, *p* = 50.5% from the prevalence of IPV in Ethiopia from the systematic review of IPV reports of the Ethiopian Demographic and Health Surveys (EDHS) [37].

### Sampling procedure and technique

**Fig. 1:**
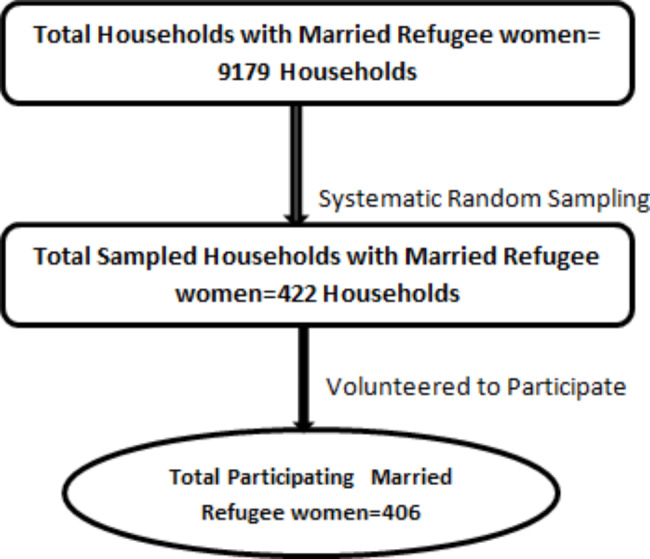
Diagrammatic presentation of the sampling procedure for the study

The list of all household units in the camp fulfilling the inclusion criteria was developed and the systematic random sampling technique was applied to select the study participants from the list. Households with married women who lived with their partners in the year preceding the study period were included. Women who were never married, and who stayed divorced or separated for over a year before the data collection period were excluded from the study(Fig. 1).

### Operational definitions

#### Intimate partner violence

IPV was defined as self-reported physical and/or sexual and/or emotional violence and/or application of controlling behavior by her husband during the 12 months before the survey [18].

#### Physical violence

if the answer to one of the following questions was affirmative: that she had been exposed to threats of physical violence such as slapping, hitting, kicking and beating; pushed, choked, or had something thrown at her; hit with something that caused/could have caused the physical injury; threatened with a knife/gun/or another object by her husband [18].

#### Sexual violence

if she stated that, against her will, she had been physically forced to have sexual intercourse, threatened/frightened into having sexual intercourse, or forced to participate in other sexual acts [18].

#### Emotional violence

If she stated that her husband applied a system to control her using emotions through criticizing, frightening, embarrassing, shaming, blaming, or otherwise manipulating her [18].

#### Attitudinal Acceptance of IPV

Acceptance was measured on a set of five negative questions with responses on a scale of five from strongly disagreeing to strongly agreeing. Women who responded as agreeing or strongly agreeing to at least one of these negative questions were regarded as having acceptance of IPV [7].

### Data collection tools

A structured interviewer-administered questionnaire was used to collect the data. The data collection tool is divided into five parts, and was adopted from the EDHS questionnaire to fit the refugee situation [7]. The questionnaire for the assessment of different forms of IPV, attitude towards IPV, and family history of violence were also taken from the EDHS’s IPV assessment questionnaire. Acceptance was measured on a set of five negative questions with responses on a scale of five from strongly disagreeing to strongly agreeing. Women who responded as agreeing or strongly agreeing to at least one of these negative questions were regarded as having acceptance of IPV [7]. Questions regarding substance use among women and partners were adopted from the substance use part of the STEPS questionnaire [38].

### Data collection procedures

Data were collected by five trained senior female nurses. The training was provided by team of experts from Gambella Regional Health Bureau and Non-Governmental Organizations(NGOs) with rich experience in management and study of IPV. The data collection process was supervised by two senior nurses who had previous experience in data collection and supervision on GBV and IPV studies. The respondents were asked in a private room or in a separate location to ensure their privacy. Visual aid was used to help women locate their feelings on the Likert scale questions.

### Data quality assurance

The data collection tool was adopted from validated tools. The questionnaire was pretested and the necessary amendment was made before data collection. Comprehensive training was provided for data collectors and supervisors on the nature of the study, study setting, and participants. The supervisors checked the completeness and consistency of the filled questionnaires on daily basis to ensure data quality. The supervisors made frequent field visits and guided the data collectors. The principal investigators also appraised the data before the data analysis to verify the completeness of the collected data.

### Data analysis procedure

Data were coded and entered into Epi-data version 3.1 and exported to SPSS version 22 for analysis. Frequencies and cross-tabulations were used to check for missed values and to describe the study population with relevant variables. Descriptive analysis was used to describe data using the percentages and frequency of the respondents by socio-demographic characteristics and other relevant variables. Both bivariable and multivariable logistic regressions were fitted. Independent variables with a *p*-value of less than 0.25 in the bivariable logistic regression were entered into the multivariable logistic regression model to control for potential confounders and to identify significant factors associated with the outcome variable. Finally, the adjusted odds ratio (AOR) along with a 95% confidence interval(CI) was estimated to assess the presence of association, and a *p*-value < 0.05 was used to declare the statistical significance in the multivariable logistic regression. Adequacy of the model to fit the outcome variable with the predictors was checked using the Hosmer and Lemeshow test for goodness of fit with *p* = 0.37.

### Ethical consideration

This study was conducted in compliance with the principles of the Declaration of Helsinki. Ethical approval was obtained from the Ethics Review Committee of the College of Health Sciences, Mettu University referenced as RPG/27/2013 and issued on 3/09/2013 in Ethiopian Calendar. Permission was obtained from the Gambella branch of the Administration of Refugee and Returnees Agency (ARRA), and a formal letter was written from the ARRA to the camp administrators. Written informed consent was obtained from each participant after the provision of comprehensive information on the nature, purpose, and procedures of the study. Participants completed the questionnaire in a separate location. Anonymity and confidentiality of the participants were maintained at all levels. Participants were assured that their participation is voluntary, and they had every right to withdraw or refuse to give information at any time during the study.

## Results

### Socio-demographic characteristics of the participants

A total of 406 study participants volunteered to take part in this study making a response rate of 96.2%. The mean age of the study participants was 32.8 years with a standard deviation of ± 11 years. Most of the study participants were Nuer in ethnicity 342 (84.2%), and followers of protestant religion 249 (61.3%). Nearly half of the participants 194 (48.2%) did not attend formal education. (Table 1).

**Table 1:** Socio-demographic characteristics of married South Sudanese refugee women in Pinyudo refugee camps, Southwest Ethiopia, 2021

Variable	Response categories	Frequency	Percent
Age category	18–24	81	20.0
	25–34	188	46.2
	35–44	21	5.2
	45 and older	116	28.6
Ethnicity	Anuak	64	15.8
	Nuer	342	84.2
Religious background	Orthodox Christian	41	10.1
	Catholic	78	19.2
	Protestant	249	61.3
	Muslim	33	8.1
	Other religions	5	1.3
Educational status	No formal education	194	47.8
	Primary school	150	36.9
	Secondary school and above	62	15.3
Occupation	Housewife	329	81.1
	Employed worker	44	10.8
	Daily laborer	25	6.2
	Merchant	8	1.9
Income contribution	Less than husband	153	43.1
	About the same	149	42.0
	More than the husband	53	14.9

### Husband/partner’s characteristics

Regarding husband’s characteristics, more than half of the husbands of participating refugee women attended school 212 (52.2%), and half of them are currently unemployed 205(50.5%). In addition, half of husbands 195(48.8%) were engaged in a polygamous marriage. Concerning husband’s alcohol and substance use, 151(37.2%) of them drink every day and, 52 (12.8%) of the husbands smoke cigarette (Table 2).

**Table 2:** Husband’s characteristics of married South Sudanese refugee women in Pinyudo refugee camp, Southwest Ethiopia, 2021

Variable	Response categories	Frequency	Percent
Attend school	Yes	212	52.2
	No	194	47.8
Occupation	Student	36	8.9
	Merchant	20	4.9
	Incentive worker	61	15.0
	Unemployed	205	50.5
	Day laborer	43	20.6
Age difference	Older than her	13	37.1
	Almost similar	12	34.3
	Younger than her	10	28.6
Engagement in a polygamous marriage	Yes	195	48.0
	No/Don’t know	211	52.0
Husband drink alcohol	Every day	151	37.2
	Once or twice a week	34	8.4
	Once or twice a month	54	12.3
	Never	167	41.1
Other substance use	Yes	115	28.3
	No	291	71.7
Type of other substance used by husbands	None	291	71.6
	Cigarette	52	12.8
	Marijuana	27	6.7
	Ganja	36	8.9

### Childhood exposure and attitude towards intimate Partner violence

Regarding the family history of IPV, 148 (36.5%) of the women have witnessed their mother being hit by their father during childhood. One hundred eighty-two (44.8%) of the refugee women had a favorable attitude towards harmful traditional practices against women. Further, 189 (46.6%) of the respondents had a favorable attitude toward physical violence and 144 (35.5%) of the respondents had a favorable attitude towards sexual violence. Only 118 (29.1%) of the respondents had a favorable attitude towards overall IPV (Table 3).

**Table 3:** Childhood exposure and attitude towards IPV among married South Sudanese women in Pinyudo refugee camp, Southwest Ethiopia, 2021

Variable	Response categories	Frequency	Percent
When you were a child was your mother hit by your father?	Yes	148	36.5
	No	218	53.7
	Parents did not live together	35	8.6
	Do not know	5	1.2
As far as you know, was your (most recent) stepmother hit or beaten by her husband?	Yes	135	33.3
	No	201	49.5
	Parents did not live together	42	10.3
	Do not know	28	6.9
Was your partner himself hit or beaten regularly by someone in his family?	Yes	99	24.4
	No	208	51.2
	Do not know	99	24.4
Attitude toward harmful practice against women	Favorable	182	44.8
	Unfavorable	224	55.2
Attitude towards physical violence	Favorable	189	46.6
	Unfavorable	217	53.4
Attitude towards sexual violence	Favorable	144	35.5
	Unfavorable	262	64.5
Attitude towards IPV	Favorable	118	29.1
	Unfavorable	288	70.9

### Substance use

Regarding use of alcohol and tobacco among the respondents, 21 (5.2%) of the respondents consume alcohol daily and 37 (9.1%) currently use tobacco (Table 4).

**Table 4:** Behavioral characteristics of married South Sudanese women in Pinyudo refugee camp, Southwest Ethiopia in 2021

Variable	Response categories	Frequency	Percent
Alcohol drinking	Every day	21	5.2
	Five to six times a week	30	7.4
	1–4 days a week	39	9.6
	1–3 days per month	35	8.6
	Never	276	68.0
	Refused/no answer	5	1.2
Tobacco use	Yes	37	9.1
	No	369	90.9

### Prevalence of intimate partner violence

Almost half of the participating refugee women, 196 (48.3%) 95% CI: (43.6–53.2), experienced IPV as measured in terms of physical, sexual, and emotional violence. Considering the specific types of violence, 164(40.4%) of refugee women experienced physical violence, 144 (35.5%) faced sexual violence and 175(43.1%) experienced emotional violence. In this study, almost a quarter of refugee women 97 (23.9%) were forced to have sexual intercourse with their partner in the past 12 months. Regarding emotional violence, 91(22.4%) reported that their husband made them feel ashamed and 152(37.4%) reported that their partner ever used her children to threaten her.

### Factors associated with intimate partner violence

Age of women, age difference between husband and women, educational status of the women, educational status of husband, occupation of women, occupation of husband, income contribution, payment of bride price, husbands having a relationship outside their marriage (polygamy), husband use of a substance, women’s use of a tobacco, women’s use of alcohol, attitudinal acceptance of IPV, witnessing IPV as a child and witnessing abuse against husband were candidate variables for the multivariable logistic regression analysis. From these, seven variables remained in the final multivariable logistic regression model. In the final multivariate model, two variables remained significantly associated with IPV. Income contribution was significantly associated with IPV. Women who made less income contribution than their husbands have experienced IPV 2.4 times more likely than women who contributed more than their husbands [AOR = 2.4, 95%CI: 1.2–5.5]. Women who had acceptance of IPV were two times more likely to experience the problem than those who do not have acceptance of IPV [AOR = 2.1, 95%CI: 1.2–3.8] (Table 5).

**Table 5:** Factors associated with IPV among married South Sudanese women in Pinyudo refugee camp, Southwest Ethiopia, 2021

Variables	Variable categories	IPV status		COR	AOR
		Yes n (%)	No n (%)	(95% CI)	(95% CI)
Age category	18–24	32 (39.5)	49 (60.5)	1	1
	25–34	86 (45.7)	102 (54.3)	1.3 (0.7–2.2)	1.2 (0.5-2.0)
	35–44	11 (52.4)	10 (47.6)	1.6 (0.6–4.2)	0.7 (0.2–2.9)
	45 and older	67 (57.8)	49 (42.2)	2.1 (1.2–3.7)	1.4 (0.7–3.3)
Income contribution	Less than husband	83 (54.2)	70 (45.8)	1.9 (0.1–3.7)	2.4 (1.2–5.5)*
	About the same	62 (41.6)	87 (58.4)	1.2 (0.6–2.3)	1.3(0.6-3.0)
	More than husband	20 (37.7)	33 (62.3)	1	1
Husband’s outside relationship (Polygamy)	Yes	118(60.5)	77(39.5)	2.6(1.7–3.9)	1.3(0.6-3.0)
	No	78(37.0)	133(63.0)	1	1
Bride Price payment	Yes	145 (54.3)	122 (45.7)	2.1 (1.3–3.2)	1.1 (5.6-2.0)
	No	51 (36.7)	88 (63.3)	1	1
Women tobacco use	Yes	21(56.8)	16 (43.2)	1.4(0.7–2.8)	1.1 (0.9–1.11)
	No	175(47.4)	194(52.6)	1	1
Husband’s Substance use	Yes	87(75.7)	28(24.3)	5.1(1.4–9.2)	4.0 (0.9–10.2)
	No	109(32.0)	182(68.0)	1	1
Attitudinal acceptance of IPV	No	67 (63.2)	115 (36.8)	1	1
	Yes	143 (36.2)	81 (63.8)	3.03 (2.2–4.9)	2.1 (1.2–3.8)*

*p-value less than 0.05; IPV: Intimate Partner Violence; COR: Crude Odds Ratio; AOR: Adjusted Odds Ratio.

## Discussion

This study determined the prevalence of IPV among married South Sudanese refugee women in Ethiopia. It also identified factors contributing to the higher probability of the problem among these disadvantaged population. In this study, half of refugee women faced some form of IPV in the year preceding the study. This implies a large-scale existence of violence against refugee women superimposed on the preexisting health and social challenges. This may further complicate the multifaceted problem of refugee women in camps, and delay the recovery and rehabilitation efforts. The observed level of IPV among South Sudanese refugee women may pose a short and long-term risk to the health and social wellbeing of refugee women, children, and young adolescents through traumatizing domestic experiences.

The current finding is much higher than the prevalence of IPV among Eritrean refugees in Shimelba refugee camp in Tigray region of Ethiopia, where 25.5% of participating refugee women experienced IPV. The relatively lower prevalence in the Shimelba study may be due to the fact that only physical violence was used to measure IPV [12].

The observed prevalence of IPV among South Sudanese refugee women in Ethiopia was higher than the prevalence of the problem in the least developed countries (37%) [2]. It is also higher than reports on the prevalence of IPV among non-refugee women from 46 Low and Middle Income Countries(LMIC) where the prevalence ranged from 5-40% [39]. The prevalence of IPV in the current study is also higher than the prevalence among non-refugee women in Ethiopia, where almost 30% of ever partnered women experience IPV in the year preceding the survey [6]. This indicates the special challenge of refugee women who endure higher levels of IPV than non-refugee women. This also implies the need of further efforts to reduce the burden of IPV in humanitarian settings [8].

The current prevalence of IPV is lower than the report from a study conducted in Iran among Afghan refugee women where almost 80% of partnered refugee women experienced IPV [40]. The difference may be attributed to the conservative nature of the Afghan community and its social systems that gives little concern to globally accepted women’s rights [41]. In addition, the current prevalence is also lower than the reported prevalence of IPV among Rohingya refugees in Bangladesh where up to 72% of partnered women experienced IPV in the year before the study [10, 42]. The Rohingya crisis unfolded in a short period giving no room for humanitarian agencies to establish systems to track and prevent the IPVs. This may explain the big difference in the prevalence [43, 44].

The finding of the current study is comparable with the prevalence of IPV among Congolese refugee women in Rwanda where almost half (49%) reported experiencing physical, emotional, or sexual violence [11]. The resemblance may be due to the geographical proximity of Eastern Congo and South Sudan, making the community characteristics related and comparable.

In the current study, women who made less income contribution than their husbands experienced IPV twice more likely than women who contributed equal or more than their husbands. This finding is supported by several studies conducted in Ethiopia and abroad. A study conducted in China showed women who have less finical income than their husbands to bear two times more risk of facing IPV [45]. A study from Nepal also revealed that women who are finically dependent on their partners to be at a higher risk for physical violence by their husbands [46]. This association is also supported by a study conducted in Hosanna, where economically independent women were less likely to face IPV [47]. This finding shows the importance of economic dependency as an enabling factor of IPV. In addition, this finding points on an important area of intervention to curb the problem through economic empowerment of refugee women. The economic dependency of married women may limit the possibility of women to separate from a violent husband and live an independent life, which increases the risk of IPV [48].

Contrarily, a recent study among Somali refugees in Ethiopia revealed an increased likelihood of IPV among economically independent women [27]. A study in India also showed the increased probability of economically empowered women experiencing IPV [49]. According to an additional study in India, change in employment pattern of women and husband were found to worsen the risk of IPV. Women who were unemployed at one visit and began employment by the next visit had an 80% higher odds of violence, as compared to women who maintained their unemployed status. Similarly, women whose husbands had stable employment at one visit and newly had difficulty with employment had 1.7 times the odds of violence, as compared to women whose husbands maintained their stable employment [50]. The increased risk of empowered and economically independent women to experience IPV may be due to romantic jealousy of their husbands [27]. Further studies are needed to boost our understanding of the dynamics in which economic independence prevents IPV, and in some instances worsen the problem.

In this study, women who have attitudinal acceptance towards IPV are twice more likely to experience IPV than their counterparts. A study among Rohingya refugees indicated a high magnitude of normalization of wife beating and association of such beliefs with IPV [51]. In addition, a study among refugee women in Australia also showed the importance of attitude on gender roles as an important factor for experiencing IPV [52]. Attitudinal acceptance entails normalization and tolerance of abusive behaviors of the intimate partner. Woman with attitudinal acceptance of IPV and normalization of partner abuse may signal the message of weakness, helplessness and impunity. Women who have attitudinal normalization of violence blame themselves and are less likely to report the incidents of violence to authorities [52].

### Limitation

This study was conducted using only quantitative methods, and it did not account for some unquantifiable experiences of refugee women. In addition, the experience of IPV was assessed for the last 12 months. This may have underestimated the actual occurrence of abusive events through recall bias. Due to legal reasons, the study included women with age 18 and above. This may have missed some important aspects of IPV related information among married refugee women aged 15–17. Further, this study did not measure the severity of IPV among married refugee women who sustained such violence. Finally, all the responses were based on the women’s self-report, memory and truthfulness in answering the questions.

## Conclusions

The current study found an alarming prevalence of IPV among South Sudanese refugee women in Ethiopia. Almost half of partnered refugee women experienced some form of IPV over the 12 months before data collection. The observed prevalence is far higher than the prevalence of IPV in the non-refugee population and warrants the need for special focus on these disadvantaged populations. Income contribution and the women’s attitudinal acceptance of IPV were associated with a higher probability of experiencing IPV among South Sudanese refugee women in Ethiopia.

### Recommendations

The widespread occurrence of IPV among refugee women warrants the need for concerted efforts from all stakeholders to combat the problem. The government of Ethiopia, ARRA, UNHCR, and other non-governmental organizations should intensify the implementation of GBV-oriented programs with an emphasis on IPV. Women empowerment has to be prioritized in general. Further studies has to be conducted to boost our understanding of the impact of economic empowerment in reducing IPV and worsening it under some circumstances. Refugee women have to be enrolled in behavior change communication programs to change the prevailing attitudinal acceptance of different forms of IPV against women. Further studies have to be conducted among other South Sudanese refugee camps in Ethiopia to allow cross sectional analysis of the problem.

## Data Availability

All data for this research article is available and can be accessed from the corresponding author at any time.

## List of Abbreviations

ARRA: Administration of Refugee and Returnees Agency
AOR: Adjusted Odds ratio
CI: Confidence Interval
EDHS: Ethiopian Demographic and Health Survey
GBV: Gender Based Violence
HIV: Human immune Virus
IPV: Intimate Partner Violence
LMIC: Low and Middle-Income Countries
SDG: Sustainable Development Goal
SGBV: Sexual and Gender Based Violence
VAW: Violence against Women
UNHCR: United Nations Higher Commissioner for Refugees
UN: United Nations
WHO: World Health Organization
